# A rare primary mediastinal mesenchymal tumor diagnosed by endobronchial ultrasound (EBUS)

**DOI:** 10.1186/s12890-026-04103-7

**Published:** 2026-01-14

**Authors:** Serap Argun Baris, Huseyin Kaya, Gozde Selvi Guldiken, Gozde Oksuzler Kizilbay, Cigdem Vural, Ilknur Basyigit, Hasim Boyaci

**Affiliations:** 1 Department of Pulmonary Medicine, Kocaeli University Faculty of Medicine Hospital, Kocaeli, Turkey; 2 Department of Pulmonary Medicine, Kocaeli City Hospital, Kocaeli, Turkey; 3 Department of Pulmonary Medicine, Canakkale Mehmet Akif Ersoy State Hospital, Canakkale, Turkey; 4 Department of Pulmonary Medicine, Kiziltepe State Hospital, Mardin, Turkey; 5 Department of Pathology, Kocaeli University Faculty of Medicine Hospital, Kocaeli, Turkey

**Keywords:** EBUS, Malignant mesenchymal tumor, Mediastinal mesenchymal neoplasm, Undifferentiated neoplasm

## Abstract

Primary mesenchymal tumors of the mediastinum are extremely rare and often pose considerable diagnostic challenges owing to their histological diversity and anatomical location. We report the case of a 51-year-old man with a 45 pack-year smoking history who was referred for evaluation of a mediastinal mass initially detected by chest radiography. Thoracic computed tomography (CT) and positron emission tomography-CT revealed a 3 × 3 cm lesion extending from the right upper and lower paratracheal regions into the right upper lobe parenchyma, demonstrating intense FDG uptake. Fiber-optic bronchoscopy revealed no endobronchial lesions. A CT-guided transthoracic needle biopsy was non-diagnostic. Subsequently, linear endobronchial ultrasound (L-EBUS) was performed, and a transbronchial needle biopsy sample was obtained for histopathological examination. Histopathological examination revealed an undifferentiated malignant mesenchymal tumor, positive for vimentin, with a Ki-67 proliferation index of approximately 30%. Despite an extensive immunohistochemical panel, no specific line of differentiation was established. Following the diagnosis, the patient was discussed at a multidisciplinary tumor board and subsequently referred to medical oncology for systemic treatment. This case highlights the role of L-EBUS in obtaining adequate tissue for the diagnosis of a rare malignant mesenchymal mediastinal tumor, particularly after inconclusive conventional biopsy attempts. Accurate diagnosis of such rare tumors requires a multidisciplinary strategy incorporating imaging, histopathology, and immunohistochemistry to guide appropriate treatment planning and prognostication of the disease.

## Introduction

Mesenchymal tumors of the mediastinum are exceedingly rare, accounting for only approximately 2–6% of all mediastinal neoplasms [1]. Except for certain exceptions, mediastinal soft tissue tumors display histological and molecular features largely analogous to those of their counterparts in other organs. With the exception of organ-specific neoplasms (e.g., gastrointestinal stromal tumors [GIST]), virtually every type of mesenchymal lesion may arise in the mediastinum [2].

In this case report, we present a case of a rare malignant mesenchymal mediastinal tumor diagnosed by linear endobronchial ultrasound (L-EBUS).

## Case report

A 51-year-old man presented to an external center with cough for several days. A suspicious opacity seen on posteroanterior chest radiography prompted contrast-enhanced thoracic computed tomography (CT), which revealed a mediastinal mass, and the patient was referred to our institution. His medical history was unremarkable for chronic disease, environmental exposures, or regular medications; however, he had a 45 pack-year smoking history.

**Fig. 1:**
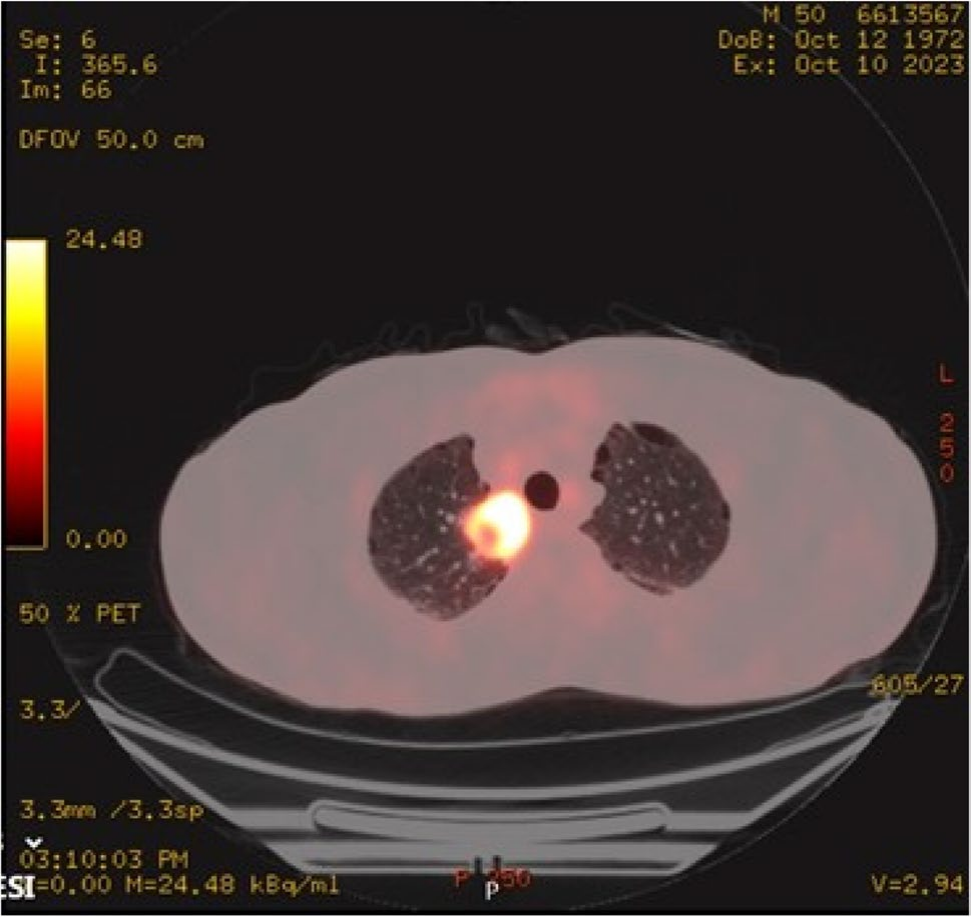
PET-CT imaging: A roughly 3 × 3 cm mass lesion extending from the right upper and lower paratracheal regions into the right upper lobe parenchyma, demonstrating FDG uptake

Positron emission tomography–CT (PET-CT) revealed a 3 × 3 cm lesion with cystic and nonmetabolic components extending from the right upper and lower paratracheal regions into the right upper lobe parenchyma (SUVmax 23.8) (Fig. 1). Additionally, a subcentimetric left upper paratracheal lymph node (SUVmax 6.8) and a subcarinal lymph node measuring 13 × 17 mm (SUVmax 7.6) were detected, both considered compatible with metastatic disease. Fiber optic bronchoscopy revealed no endobronchial lesions. CT-guided tru-cut biopsy of the right upper lobe lesion demonstrated inflamed lung parenchyma with focal necrosis, without definitive evidence of malignancy. Focal nuclear staining for TTF-1 and p40 was considered non-diagnostic. The pathology report recommended repeat sampling.

**Fig. 2:**
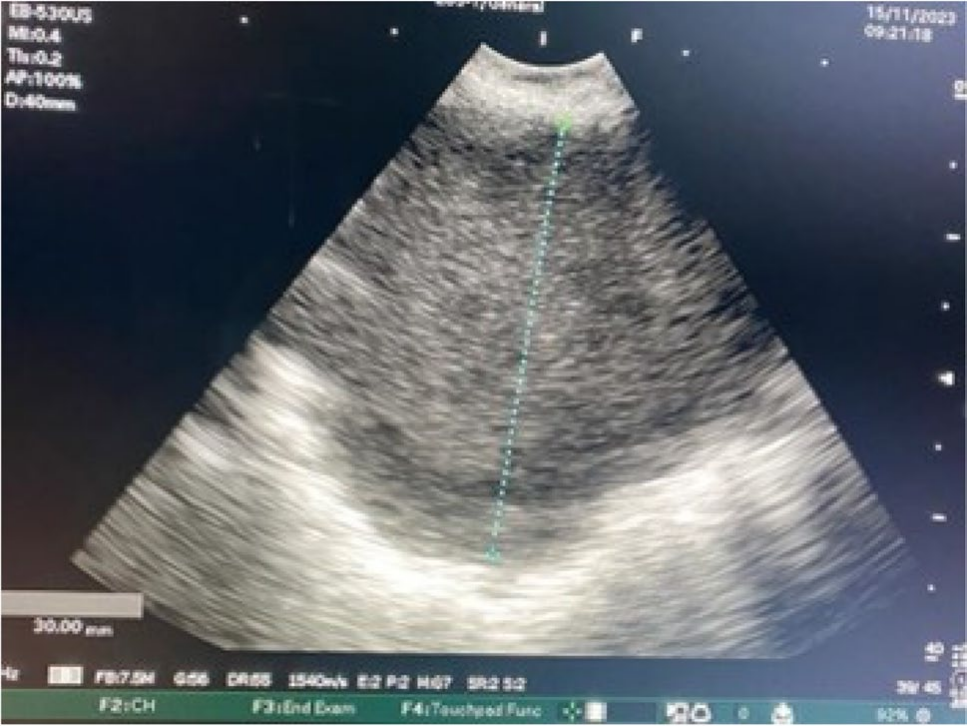
EBUS imaging: A right paratracheal lesion measuring approximately 3 × 3 cm, with heterogeneous echo texture and irregular margins

**Fig. 3:**
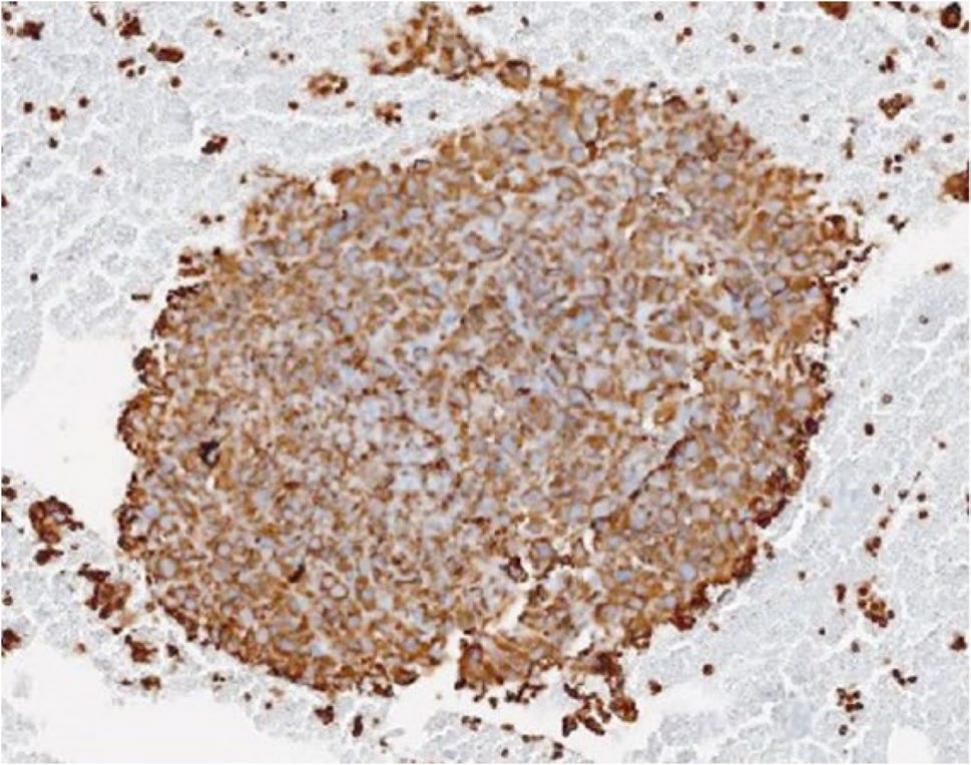
On vimentin immunostaining, the tumor tissue exhibits diffuse and strong cytoplasmic positivity

Given these non-diagnostic findings, linear EBUS-TBNA was performed. The procedure was performed using a Fujifilm linear EBUS system (EP-6000 processor with SU-1 ultrasound unit), and tissue sampling was performed using a 22-gauge SonoTip EBUS Pro needle (Medi-Globe). L-EBUS examination revealed a right paratracheal lesion measuring approximately 3 × 3 cm, characterized by heterogeneous echotexture, mixed echogenicity, irregular margins, and focal cystic areas without detectable vascularity. The lesion was best visualized between stations 2R and 4R, which are the closest anatomical locations to the mass (Fig. 2). Additional lymph nodes were also observed in the subcarinal station (12 mm), station 11R (5 mm), station 2 L (8 mm) and station 10 L (4 mm). Transbronchial needle biopsy was subsequently performed from the right paratracheal lesion, subcarinal lymph node, and left upper paratracheal lymph node.Three sampling passes were performed, and during each pass, the needle was advanced and withdrawn approximately 15–20 times to obtain adequate material. No procedure-related complications or bleeding were noted. A histological specimen was obtained via EBUS-TBNA, and a cell block was prepared for further analysis. An extensive immunohistochemical panel was applied, including epithelial (TTF-1, p40, p63, pancytokeratin, CK5/6, EMA), neuroendocrine (synaptophysin), melanocytic markers (Melan-A), myogenic markers (SMA, desmin, myogenin), and vascular markers (CD34), and additional lineage-associated markers. All lineage-specific markers were negative, while vimentin showed diffuse and strong cytoplasmic positivity. Morphologically, the tumor consisted of pleomorphic malignant cells. The combined morphological features and immunoprofile supported the diagnosis of an undifferentiated malignant mesenchymal tumor. The Ki-67 proliferation index was approximately 30% (Fig. 3). Histopathological evaluation of the lymph node samples was also consistent with metastatic involvement.

The patient was subsequently evaluated by an institutional multidisciplinary tumor board and was classified as inoperable. Therefore, surgical resection was not recommended. The patient was referred to the medical oncology department, where he received six cycles of adriamycin and ifosfamide chemotherapy. Follow-up assessments were continued in the medical oncology department.

## Discussion

Although the majority of mediastinal tumors are of epithelial origin, mesenchymal neoplasms, often representing metastatic disease, also occur [3]. These tumors arise from a heterogeneous array of cell lineages, including adipocytes, fibroblasts, myofibroblasts, vascular structures, skeletal muscle cells, and peripheral nerve sheath components, or may exhibit undifferentiated morphology. Approximately half of the primary mesenchymal mediastinal neoplasms are reported to be malignant [4]. Common mediastinal mesenchymal tumors include neurogenic tumors, liposarcomas, solitary fibrous tumors, and synovial sarcoma [5].

Primary malignant mesenchymal tumors of the mediastinum are exceedingly rare, with only a limited number of cases described in the literature. This rarity contributes to the difficulty in diagnosis, necessitating careful pathological and radiological correlation. According to the World Health Organization (WHO) classification of soft tissue tumors, undifferentiated sarcomas constitute a distinct category, and several of these entities have occasionally been reported in the mediastinum. Tumors such as angiomatoid fibrous histiocytoma, ossifying fibromyxoid tumor, myoepithelioma, alveolar soft part sarcoma, clear cell sarcoma, and synovial sarcoma do not arise from normal mediastinal tissues but exhibit characteristic histological patterns associated with specific genetic alterations. Notably, most of these tumors have been documented in only a small number of mediastinal cases [6].

The accurate diagnosis of primary mediastinal mesenchymal neoplasms is critical for guiding therapy and prognostication. In cases of suspected malignant mediastinal tumors, imaging modalities such as CT, MRI, or PET/CT may be selectively utilized based on the clinical context, pretest probability, and multidisciplinary assessment, rather than being applied routinely. Histopathological diagnosis of mediastinal masses using traditional methods is often invasive and carries additional risks. However, EBUS allows for real-time, minimally invasive sampling of mediastinal masses [7]. This method generally offers high diagnostic yield and is a safer alternative for patients [8].Although transbronchial forceps biopsy may further aid in the characterization of mediastinal masses, it was not performed in our case due to the lesion’s proximity to major mediastinal vessels, and the potential risk of bleeding.

In conclusion, mediastinal mesenchymal tumours are quite rare, and the diagnostic process is often difficult. In these cases, reaching a definitive diagnosis requires the combined evaluation of the tumour’s morphological, immunohistochemical, and imaging findings. In this case, the diagnosis made using L-EBUS once again highlights the value of minimally invasive methods in rare mesenchymal tumours. The selection of an appropriate method in the diagnostic process is of great importance in terms of reducing invasiveness and providing a rapid and accurate approach that affects prognosis.

## Data Availability

All relevant data are included in this published article. Additional details are available from the corresponding author upon reasonable request.
